# Microbial Metabolic Potential of Phenol Degradation in Wastewater Treatment Plant of Crude Oil Refinery: Analysis of Metagenomes and Characterization of Isolates

**DOI:** 10.3390/microorganisms8050652

**Published:** 2020-04-30

**Authors:** Signe Viggor, Merike Jõesaar, Pedro Soares-Castro, Tanel Ilmjärv, Pedro M. Santos, Atya Kapley, Maia Kivisaar

**Affiliations:** 1 Institute of Molecular and Cell Biology, University of Tartu, 23 Riia Street, 51010 Tartu, Estonia; merike.joesaar@ut.ee (M.J.); tanel.ilmjarv@ut.ee (T.I.); maia.kivisaar@ut.ee (M.K.); 2 Centre of Molecular and Environmental Biology (CBMA), University of Minho, Campus de Gualtar, 4710-057 Braga, Portugal; pcastro@bio.uminho.pt (P.S.-C.); psantos@bio.uminho.pt (P.M.S.); 3 Director’s Research Division, CSIR-National Environmental Engineering Research Institute (CSIR-NEERI), Nehru Marg, Nagpur 440 020, India; a_kapley@neeri.res.in

**Keywords:** phenol, bacterial community, oil refinery wastewater, metagenome, *Pseudomonas*, *Acinetobacter*

## Abstract

The drilling, processing and transportation of oil are the main sources of pollution in water and soil. The current work analyzes the microbial diversity and aromatic compounds degradation potential in the metagenomes of communities in the wastewater treatment plant (WWTP) of a crude oil refinery. By focusing on the degradation of phenol, we observed the involvement of diverse indigenous microbial communities at different steps of the WWTP. The anaerobic bacterial and archaeal genera were replaced by aerobic and facultative anaerobic bacteria through the biological treatment processes. The phyla Proteobacteria, Bacteroidetes and Planctomycetes were dominating at different stages of the treatment. Most of the established protein sequences of the phenol degradation key enzymes belonged to bacteria from the class Alphaproteobacteria. From 35 isolated strains, 14 were able to grow on aromatic compounds, whereas several phenolic compound-degrading strains also degraded aliphatic hydrocarbons. Two strains, *Acinetobacter venetianus* ICP1 and *Pseudomonas oleovorans* ICTN13, were able to degrade various aromatic and aliphatic pollutants and were further characterized by whole genome sequencing and cultivation experiments in the presence of phenol to ascertain their metabolic capacity in phenol degradation. When grown alone, the intermediates of catechol degradation, the meta or ortho pathways, accumulated into the growth environment of these strains. In the mixed cultures of the strains ICP1 and ICTN13, phenol was degraded via cooperation, in which the strain ICP1 was responsible for the adherence of cells and ICTN13 diminished the accumulation of toxic intermediates.

## 1. Introduction

Crude oil refineries are large facilities that transform crude oil into different useful products such as gasoline, diesel fuel, asphalt, heating oil and liquefied gas, among others. The processing of oil consumes a large amount of water (cooling, cleaning, etc.). Part of it can be recycled but due to the leakages, the rest of it has to be cleaned. Wastewater formed during the valuation of crude oil in the desalting, dehydration, condensation and fractionation processes is contaminated with various types of recalcitrant aromatic and aliphatic hydrocarbons [1,2]. The content of the wastewater depends on the refinery configuration, operation procedures and type of oil being processed [3]. In the areas where water resources are limited, the reuse of industrial wastewater in the process or in other applications (irrigation of the green areas, fire control, etc.) is desirable [4].

The treatment of the wastewater is usually a multistage process involving physical, chemical and biological methods. The treatment process in the wastewater treatment plant (WWTP) of the refinery (Uran, India) studied in the current work includes mechanical oil separation, the addition of nutrients (urea and (NH_4_)_2_HPO_4_) to enhance the degradation of the dissolved and colloidal organic matter, biological treatment in a bio-tower (a vertical tank filled with high-surface area polypropylene elements on what the biofilm of pollutants-degrading microorganisms are formed), the removal of washed out biomass from treated water in a clarifier unit by gravity and the final treatment is conducted in an aerated guard pond. A regular determination of the efficiency of the treatment is followed (oil content, total suspended solids, biological and chemical oxygen demand) to meet the established requirements for the cleaned water released into the environment [5].

The list of the pollutants found in refinery wastewater is long, and it contains simple and complex hydrocarbons [6,7]. Indigenous microbial communities of bacteria and archaea are capable of using a wide range of hydrocarbons as nutritional resources either aerobically or anaerobically [6,7,8,9]. The proportion of mono- and polyaromatic compounds is usually lower than alkanes but they are highly toxic to living organisms, even at very low concentrations. Phenols are one of the simplest aromatic compounds that are highly soluble in water, which are found in the wastewater of refinery (0.1 to 5 mM), coal processing (up to 72 mM) and petrochemical plants (up to 13 mM), and in the pharmaceutical, plastic, paper, etc., industries (up to 17 mM) [2,10,11]. Therefore, it is important to remove these contaminants from industrial effluents before their discharge into the environment. Biodegradation of aromatic compounds is limited by the difficulties in cleavage of highly stable benzene rings. Several microorganisms have acquired the relevant enzymes and are able to tolerate phenol and use it as a source of carbon and energy. Aerobic degradation of phenol is usually initiated by single or multicomponent phenol hydroxylases (PHs) that results in the formation of catechol, which is further cleaved either via the catechol meta pathway by catechol 2,3-dioxygenase (C23O) or via the catechol ortho pathway by catechol 1,2-dioxygenase (C12O).

Several culture-dependent and independent analyses have identified diverse and complex communities possessing the catabolic genes responsible for the degradation of oil hydrocarbons in various conditions [6,9,12]. WWTPs are hotspots for the highly specialized microorganisms that are able to degrade the pollutants found in the influent. The isolation and characterization of such strains and monitoring their performance in bioaugmentation experiments could have practical values in the enhancement of the degradation of pollutants. The current work was conducted to gain an insight into the indigenous microbial communities at the different treatment steps of a WWTP of a crude oil refinery and the diversity of genes involved in the degradation of phenol, based on the culture-independent metagenomic approach. The second part of the work evaluates the diversity of the bacterial strains isolated from a WWTP and their ability to degrade aromatic compounds, with a special focus on two phenol-degrading strains. It is important to mention that these two strains are also able to utilize aliphatic hydrocarbons, and therefore they have a high potential to be applicable in the enhancement of the bioremediation of crude oil pollution.

## 2. Materials and Methods

### 2.1. Sampling

Samples from the wastewater treatment plant (WWTP) of an Indian crude oil refinery were collected at the different steps of the treatment process: from the surge pond sludge (S), from the clarifier outlet (C) and from the guard pond outlet (G), in January 2016. Separate subsamples were taken in triplicates for the DNA- (the samples S, C and G) and cultivation-based (the samples S and C) analysis. In January, the temperature’s average maximum and minimum values were 36 °C and 21 °C, respectively.

### 2.2. Shotgun Metagenomics

The genomic DNA was extracted from the sampled triplicates of S, C and G with the FastDNA™ Spin Kit for Soil (MP Biomedicals, LLC, Irvine, CA, USA), according to the manufacturer’s instructions. The genomic DNA of the microbial communities was processed according to the Illumina (Illumina, San Diego, CA, USA) instructions to generate Nextera XT paired-end libraries and was sequenced using the Illumina NextSeq500 platform (2 × 150 bp), at Instituto Gulbenkian de Ciência, Portugal. A description of the analysis of the sequences and an overview of the assembly statistics are given in Table S1.

### 2.3. Prokaryotic Taxonomic Assignment of Shotgun Metagenomics Datasets

The structure of the microbial communities represented in the shotgun metagenomics datasets was assessed with Metaxa2 [13], by mapping the filtered paired-reads to the SILVA database of SSU rRNAs, v. 128 [14] (Table S2). Alpha-diversity indices were estimated with the “diversity” function of the package vegan v. 2.5-2 [15], see Table S3.

### 2.4. Identifying CDS Coding for Protein Homologs of the C12O, C23O and PH Families

The protein sequences of the putative hydrocarbon oxygenases—homologs of the members of the C12O, C23O and PH families—were identified in the assembled contigs by BLASTP. The query amino acid (aa) sequences were obtained by aligning the representative members of each protein family, retrieved from the different bacterial genera (33 C12O homologs; 44 C23O homologs; 40 PH homologs) with MAFFT v. 7 [16]. Consensus regions were estimated from the alignments and the respective sequences, including aa residues common to at least 50% of the aligned proteins, were extracted and used in the BLASTP homology search (Table S4). Only BLASTP hits with an e-value of <0.1, aa identity of >40% and query coverage of >50% were considered putative homologs of hydrocarbon oxygenases (Table S5). From the set of the identified putative homologs, representative sequences of the blast hits (protein species) were selected and used as the operational protein families (hereby designated as OPFs) (Table S6). The maximum likelihood midpoint rooted homology trees were generated for each protein family with the respective OPFs, by using PhyML v. 3.0 [17], with 1000 bootstrap sets, the LG as the best-fit model (evaluated by the ProtTest tool [18]), the kappa estimated, 4 substitution rate categories, the gamma distribution parameter estimated, a BIONJ starting tree, with an optimization of the topology, branch lengths and the rate parameter.

### 2.5. Isolation and Identification of Strains

The direct selection of the bacteria that are able to degrade petroleum hydrocarbons was done from plated-out cultures of the collected surge pond sludge and clarifier outlet samples, taken in January 2016 on selective M9 minimal agar plates, containing phenol (1.3 mM) or crude oil (Lukoil Oil Company OAO, Moscow, Russia; vapour phase), as the only C-source. Samples were also plated onto low-nutrient solid R2A plates (Difco, Sparks, MD, USA), without any additional substrates. In addition, to isolate the salt-tolerant (up to 20% of the NaCl concentration) bacterial strains, the samples were plated onto SM1 [19] and HMC [20] agar plates or minimal HMD [20] agar plates supplemented with the same growth substrates that were used in the M9 plates. The plates were sealed and incubated at 30 °C for 7 days. The carbon source utilization tests of the purified and morphologically different bacteria (Table S7) were performed on the minimal media supplemented with phenol (2.5 mM), Na-salicylate (1.3 mM), toluene (vapour), and *m*-, *p*- and *o*-cresol (all 1.3 mM). All the chemicals were purchased from Sigma-Aldrich (Steinheim, Germany). Pure bacterial cultures were stored in 20% glycerol at −80 °C.

### 2.6. Sequencing and Analysis of the Genomes of the Isolated Strains Acinetobacter venetianus ICP1 and Pseudomonas oleovorans ICTN13

The extraction of the genomic DNA of the strains ICP1 and ICTN13 was performed with the JETquick DNA Spin Kit (Genomed GmbH, Löhne, Germany) according to the manufacturer’s protocol. The DNA was sequenced by Instituto Gulbenkian de Ciência in Portugal, with the Illumina MiSeq platform using the Nextera XT protocol for the library creation. The details of the followed pipeline for the genome assembly, annotation and analysis are provided in Supplementary Data S1.

### 2.7. Cultivation of Bacteria on Phenol Liquid Medium

The bacterial strains were grown overnight on an R2A medium supplemented with 1.3 mM phenol at 30 °C. The bacterial cells were separated by centrifugation (1700× *g*, 10 min), resuspended in a minimal medium and used for inoculation of the fresh minimal medium containing 1.3 mM phenol. Two-hundred-and-fifty mL Erlenmeyer flasks containing 50 mL of the growth medium were incubated on a rotary shaker (100 rpm) at different temperatures. Three independent experiments were conducted with three technical replicates. The growth of the strains was determined spectrophotometrically at 580 nm. The growth rate and the length of the lag phase (lag-time) of the strains ICP1 and ICTN13 grown on the phenol-containing minimal medium were calculated using the modified Gompertz model, as described by Viggor et al. (2008) [21]. The samples taken for the chemical analyses were centrifuged at 12,000× *g* for 1 min and the supernatants were kept at −20 °C until the analysis.

### 2.8. Chemical Analyses

The concentrations of phenol and catechol were determined using an Agilent Technologies (USA) 1200 Series high-performance liquid chromatography (HPLC) system, with a quaternary pump, an autosampler and a 5-channel variable wavelength UV-Vis detector, as described by Jõesaar et al. (2017) [22]. The concentration of 2-hydroxymuconic semialdehyde in the cell-free growth medium was determined spectrophotometrically at 375 nm.

### 2.9. Determination of Specific Activities of Enzymes

The specific activities of C12O and C23O were determined by following the formation of the corresponding intermediates at 260 and 375 nm, respectively [23]. The protein concentration was determined spectrophotometrically by using bovine serum albumin as a standard [24].

### 2.10. GFP-Tagging of Strains ICTN13 and ICP1

To label the *P. oleovorans* strain ICTN13 and the *A. venetianus* strain ICP1 with the *gfp* marker, the *gfp*-delivery plasmid pBK-miniTn7-*gfp*, containing a gentamicin-resistance marker, was used [25,26]. The *gfp*-delivery plasmid was transferred from *Escherichia coli* CC118λpir (Table S8) into the strains ICTN13 and ICP1 by conjugation using the *E. coli* strain HB101 as a host of the conjugative helper plasmid pRK2013 and the *E. coli* strain AKN68-carrying helper plasmid pUXBF13, which is necessary for mini-Tn*7* transposition in *trans* (Table S8). Transconjugants were selected on agar plates supplemented with hexadecane vapour and gentamicin (10 μg mL^−1^), and were examined for the expression of the *gfp* with the fluorescence microscope Olympus BX41 (excitation and emission wavelengths were 480 and 500 nm, respectively). The obtained strain ICTN13-GFP expressed the *gfp*, while ICP1-GM exhibited only gentamicin resistance (no fluorescence of the *gfp* was detected). The conjugation experiments were carried out in triplicate.

### 2.11. Flow Cytometry Analysis

The bacterial populations were analyzed using the flow cytometer FACSAria I (BD Biosciences, 2350 Qume Drive, San Jose, CA, USA). Each measurement involved the analyses of 30,000 events. For these experiments, the wild type strains were labelled with the *gfp* marker by using the *gfp*-delivery plasmid pBK-miniTn7-gfp, containing a gentamicin-resistance marker. As only the strain ICTN13-GFP expressed the *gfp*, whereas ICP1-GM exhibited only gentamicin resistance, this allowed us to distinguish the cells of these strains by a fluorescence-activated cell sorting (FACS) analysis. The samples were taken from the populations of the single strains (ICP1-GM and ICTN13-GFP) and the mixed populations grown on the minimal medium supplemented with 1.3 mM phenol at 30 °C. For the fluorochrome excitation, a laser with a wavelength of 488 nm and a nominal power of 20 mW was used. Fluorescence was detected using a 530/30 nm optical filter. At each time point, three parallel measurements from one sample were conducted and together three independent cultivations were performed. In the calculations of the proportion of the single strains in the mixture, the ratio of the non-fluorescent/fluorescent cells of the strain ICTN13 in the single strain cultivation was taken into account. The data presented in the figures are with a standard deviation lower than 14%.

### 2.12. Effect of Higher Phenol Concentrations on the Growth of the Strains

The strains ICP1 and ICTN13 and their mixed cultures were grown on a minimal medium supplemented with different concentrations of phenol (0–10 mM) at 30 °C in a multi-well microplate (200 μL; 800 rpm). The absorbance at 580 nm was measured after 24 and 48 h with a microplate reader (Tecan Sunrise-Basic, Tecan Trading AG, Switzerland). Each assay was performed three times with at least 3 parallels for each phenol concentration.

The data presented in the figures are with a standard deviation lower than 15%.

### 2.13. Biofilm Formation Assay in Multi-Well Plates

For the determination of the biofilm formation, the Fletsher’s method was used [27], with some modifications to the previously described method by Jakovleva et al. (2012) [28]. A 100 μL volume of the minimal medium supplemented with phenol (1.3 mM) was placed on a well of polystyrene multi-well plates with a hydrophobic coating (Cellstar, Greiner Bio One International GmbH, Austria) and inoculated with 1 μL overnight-grown bacterial cultures of the strains ICTN13 or ICP1, or mixed cultures of these strains. The following steps were performed according to the procedure by Jakovleva et al. (2012) [28]. The biofilm development was measured after 24, 48 and 72 h. Each assay was performed at least three times with 16 parallel experiments for each variant.

### 2.14. Data Availability

The sequences derived from the shotgun metagenomics approach with Illumina NextSeq500 are available in the NCBI database with the BioProject database ID PRJNA574406, associated with the SRA accession numbers ranging from SRR10190793 to SRR10190799.

## 3. Results and Discussion

### 3.1. Changes in Composition of the Bacterial Communities in the Different Steps of the Crude Oil Refinery WWTP

The composition of the crude oil-degrading microbial communities in the polluted area changes with time as it is affected by the particular environmental conditions, oil content and composition. In the current work, we analyzed the composition of bacterial communities in the wastewater treatment plant (WWTP) of an Indian crude oil refinery. The samples for the DNA-based methods were taken from three sites of the WWTP: the surge pond (S), the clarifier (C) and the guard pond (G). The surge pond is a place where all the liquid waste (wastewater and remaining oil) produced in the refinery is collected. The excess oil is subjected to a recovery process and the remainder is treated in a bio-tower via a biofilm process. The outlet of the bio-tower is directed to the clarifier to remove the washed-out biomass (part of the biofilm formed on plastic carriers). The aerated guard pond is the last biological treatment step before the cleaned water is led into the environment.

**Figure 1:**
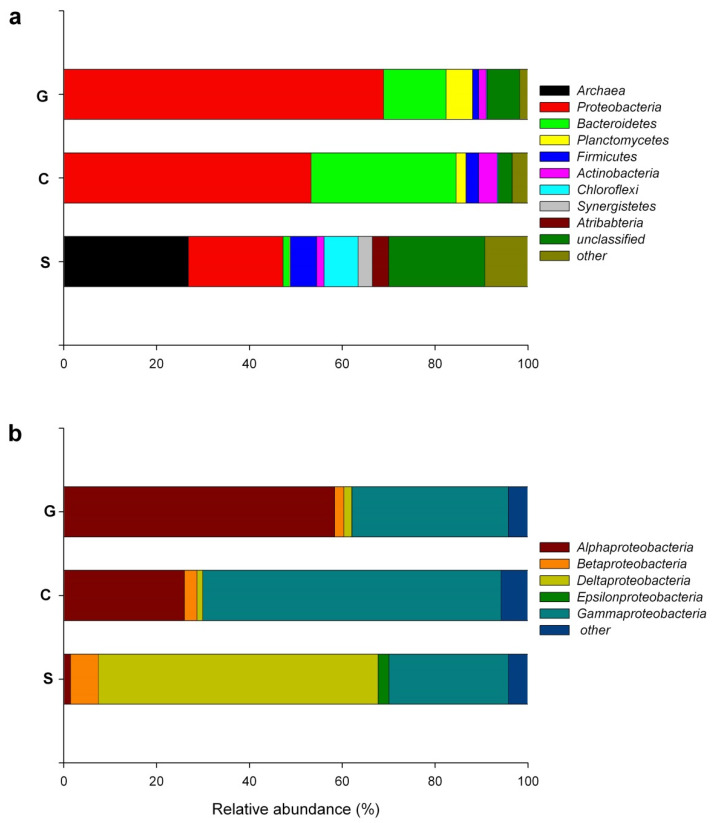
Distribution of 10 phyla and domain Archaea (**a**), and classes of the phylum Proteobacteria (**b**) in the surge pond (S), the clarifier (C) and the guard pond (G) samples (>3% relative abundance of bacteria).

The first analysis of the communities of the studied samples using 16S-metabarcoding has been published previously in the paper by Soares-Castro et al. (2019), which focused on the role of the seasonal effect to the diversity of the bacterial communities involved in the nitrogen cycle, considering also the effect of the chemical parameters (chemical oxygen demand (COD) and concentration of nitrogen compounds) of the samples [29]. The current study used shotgun metagenomics datasets to characterize the diversity of the microbes that are able to degrade aromatic compounds in the samples taken during the winter. Shortly after, the analysis of the sequences in the shotgun metagenomics datasets (Figure 1; Table S2) revealed the high distribution of strictly anaerobic bacterial phyla, for example Chloroflexi (class Anaerolineae), Atribacteria, Acetothermia, Synergistes (genera *Thermovirga*, *Anaerobaculum*) and Firmicutes (class Clostridia), in the surge pond sample. These results are in accordance with the results of Soares-Castro et al. (2019) [29]. In aerobic conditions (the samples C and G), the abundance of these phyla decreased significantly. At the same time, the proportion of the phylum Proteobacteria increased from 23%, estimated in sample S, up to 61% in sample G. The reads closely related to the sequences of the domain Archaea (28%) were dominating in sample S (classes Methanobacteria and Methanomicrobia), while in samples C and G, they were represented only at a very low degree (0.25% and 0.05%, respectively) (Figure 1a; Table S2). Such variation in the abundance of Archaea is probably caused by the changes in the oxygen concentrations throughout the treatment process. Sarkar et al. (2016) showed that a petroleum refinery sludge (dissolved oxygen concentration 0.19 mg L^−1^) contains a versatile microbial community, dominated by anaerobic Deltaproteobacteria and Euryarchaeota [6]. Our results of the analysis of the community of the surge pond sample are in accordance with this report. One of the dominant genera within the class Deltaproteobacteria were *Smithella* and *Syntrophorhabdus*. It has been proposed that these bacteria are able to degrade alkanes and phenol under methanogenic conditions in close cooperation with Archaea [30,31]. In sample C, the class Gammaproteobacteria’s genera *Methylophaga, Marinobacter* and *Marinobacterium* had higher counts than the other genera (Table S2). Gutierrez and Aitken (2014) discovered that methylotrophic *Methylophaga* strains are also methanotrophic, which explained the bloom of these bacteria during the active phase of the Deepwater Horizon oil spill [32]. The species of the *Marinobacter* are able to degrade aliphatic and aromatic hydrocarbons and they have been frequently found in oil-polluted environments [33].

After the aerobic treatment in the bio-tower (samples C and G), the majority of the anaerobic microorganisms were replaced by aerobic and facultative anaerobic bacteria (Figure 1; Table S2). The abundance of the phyla Proteobacteria and Bacteroidetes increased approximately 2.5 and 22 times, respectively, in sample C (Figure 1a). An almost thirty times higher abundance was determined for the phylum Planctomycetes in the guard pond sample than in the surge pond sample. Such compositional changes have been described in several oil-degrading communities [9,34,35,36,37].

Relevant changes were observed also among the proportions of the classes of the phylum Proteobacteria, where the proportion of Deltaproteobacteria was minor in comparison with Gamma- and Alphaproteobacteria in samples C and G, respectively (Figure 1b). Gammaproteobacteria were always represented in a higher number. The bacteria from these classes have a versatile metabolic activity, being able to degrade aliphatic and/or aromatic hydrocarbons (Gammaproteobacteria genera *Marinobacterium*, *Marinobacter*, *Methylophaga*, *Simiduia*, *Rheinheimera*, *Pseudomonas* and *Alcanivorax;* Alphaproteobacteria *genera Roseovarius, Paracoccus, Thalassospira, Sphingomonas*) and produce biosurfactants or exopolysaccharides to enhance the bioavailability of the crude oil components or have a role in the biofilm formation [33,38,39,40,41,42].

### 3.2. Diversity of Key Catabolic Genes (PH, C12O and C23O) of Phenol Degradation in the WWTP Samples

The crude oil processed in the studied crude oil refinery originates from the deep water deposits of the eastern part of the Indian Ocean and has a low sulphur (0.17%), high wax (11%–37%), low asphaltenes, low aromatics (25%) and high alkane (60%) content [43]. The current work assessed the aromatic compounds’ (focus on phenol) biodegradation ability in the microbial community of the crude oil refinery’s WWTP. Therefore, the whole metagenomes of the samples taken from the three treatment steps, i.e., the surge pond (S), clarifier (C) and guard pond (G), were sequenced and analyzed. The protein sequences of the phenol hydroxylase (PH), catechol 2,3-dioxygenase (C23O) and catechol 1,2-dioxygenase (C12O) were identified in the assembled contigs using the reference sequences from different bacterial genera (Table S5).

**Figure 2:**
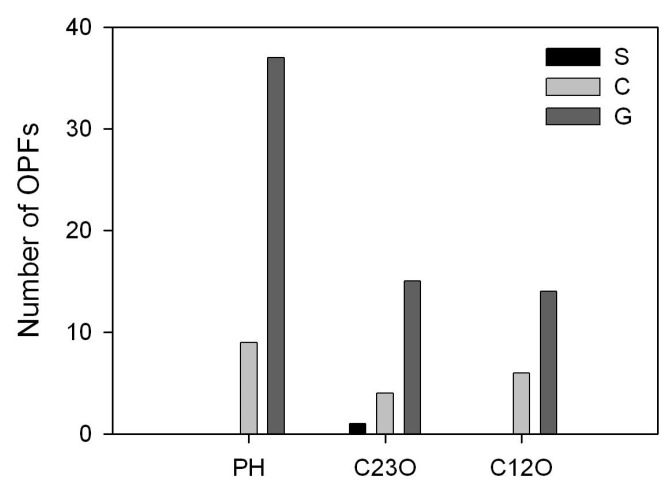
Number of the phenol hydroxylase (PH), catechol 2,3-dioxygenase (C23O) and catechol 1,2-dioxygenase (C12O) encoding the operational protein families (OPFs) detected in silico from the metagenomes of the surge pond (S), clarifier (C) and guard pond (G) samples.

The representative sequences of the investigated proteins (operative protein families (OPFs)) predicted by BLASTP (Table S6) were used to generate phylogenetic trees for each protein group (Figures S1–S3). All protein groups were detected at a higher number in samples C and G (Figure 2), revealing the enhanced biodegradation ability of the bacteria existing in the bio-tower and guard pond. The lowest number of OPFs was obtained for sample S, where only sequences of the C23O were found. Such a difference can be explained by the predominance of the anaerobic microorganisms in the surge pond sample (Figure 1) and the lack of sequences of the genes encoding the anaerobic degradation pathways of aromatic compounds in the used reference sequences database (Table S4). The BLASTP analysis of the PHs in the metagenomes of sample S detected several sequences, but as they were distant from the other found sequences and the closest reference was the copper-transporting P-type ATPase (Table S5), they were eliminated from the further analysis.

The phylogenetic analysis of the retrieved BLASTP hits of the protein sequences with an e-value of <0.1, amino acid identity of >40% and query coverage of >50% resulted in sequences not only from the three protein groups, but also several closely related homologs. In the case of the reference sequences of the different single and multicomponent phenol hydroxylases, the genes encoding the following proteins were detected in the metagenomes: multicomponent phenol hydroxylase, toluene monooxygenase, 4-hydroxybenzoate 3-monooxygenase, 2,6-dichlorophenol 6-monooxygenase, 3-(3-hydroxy-phenyl)propionate hydroxylase and pentachlorophenol monooxygenase (Figure S1). The three last proteins were determined only in the guard pond sample, while the other proteins were also found in the clarifier sample. The protein sequences retrieved from the BLASTP analysis were phylogenetically diverse, being similar to those described in Alpha-, Beta- and Gammaproteobacteria, Bacteroidetes, Planctomycetes and Actinobacteria.

C12O and C23O are responsible for the cleavage of the dihydroxylated benzene, catechol, which is the central intermediate in aromatic compounds degradation. From the three protein groups, only the sequences of the C23O were found in all the reconstructed metagenomes derived from all three samples studied (Figure S2). The detected C23Os were phylogenetically similar to the C23Os described in the bacteria from the classes Alpha-, Beta- and Gammaproteobacteria.

The sequences of the C12Os of the reference strains were used for the determination of the diversity of intradiol cleavage enzymes in the refinery’s WWTP (Figure S3). As the structure of the catalytic domain of the C12Os is similar to protocatechuate 3,4-dioxygenase, hydroquinol 1,2-dioxygenase and 6-chlorohydroxyquinol 1,2-dioxygenase [44,45], all these protein groups were identified also in the current study. The highest number of hits corresponded to the putative hydroquinol 1,2-dioxygenase of the class Alphaproteobacteria.

Although there were proteins highly similar to those found in the database, some of them clustered separately from the known sequences (Figures S1–S3). Surprisingly, the in silico analysis of the sequences of the metagenomes did not reveal the presence of any PHs, C23Os and C12Os of the well-known hydrocarbons’ degrading bacterial genera such as *Alcanivorax*, *Pseudomonas* and *Acinetobacter*, among others, but nevertheless, they were detected by Metaxa2. However, several proteins of the halotolerant marine oil hydrocarbons’ degrading genera, e.g., *Marinobacter*, *Marinobacterium*, *Sphingomonas*, *Polymorphum* and *Paracoccus*, were found in the metagenomes [33,46]. The culture-independent and culture-dependent methods usually result in quite a diverse picture of the microbial communities [47,48]. This might be the reason why we detected so many sequences distant from the cultured reference strain’s sequences.

To sum up, the distribution and diversity of the DNA sequences encoding for the aromatic hydrocarbons’ degrading proteins identified by the metagenomes of the samples taken from the different steps of refinery’s WWTP were different. Most of the established protein sequences belonged to bacteria from the class Alphaproteobacteria.

### 3.3. Diversity of Aromatic Compounds Degrading Isolates

Phenol can be found in several industrial wastewaters: oil refineries, coal processing plants, and pharmaceutical, textile and plastic industries, etc. [49]. The WWTPs receiving such wastewater are potential sources of microbes that have an enhanced degradation capability and improved tolerance to pollutants.

One of the aims of the current study was to isolate the oil hydrocarbons’ degrading bacteria with their potential application in the bioaugmentation experiments of WWTPs treating aromatic pollutants-rich wastewater. Altogether, 35 strains (from genera *Pseudomonas, Acinetobacter, Bacillus, Cellulomonas, Marinobacter, Halobacillus, Pontibacillus* and *Fulvimarina*) were isolated and identified from the surge pond and clarifier samples (Table S7). In addition to phenol, other growth substrates were selected for testing as they are also components of the crude oil (toluene, cresols, hexadecane) or they are central metabolites in the degradation of aromatic hydrocarbons (Na-salicylate).

Fourteen strains (Table S7) were able to use phenol, Na-salicylate, cresols and/or toluene as an only growth substrate and were subjected to PCR-amplification to detect the key catabolic genes encoding the catechol 2,3-dioxygenase (C23O), catechol 1,2-dioxygenase (C12O) and multicomponent phenol hydroxylase (LmPH). The majority of these strains belonged to the genus *Pseudomonas* (six strains; class Gammaproteobacteria), whereas the genera *Acinetobacter* (class Gammaproteobacteria) and *Bacillus* (phylum Firmicutes) were represented both with two and five strains, respectively, and *Cellulomonas* (phylum Actinobacteria) with one strain. Interestingly, 5 strains from 11 (i.e., the strains ICTN2, ICP1, ICTN4a, ICTN12 and ICTN13) were able to grow well also on the aliphatic hydrocarbon, hexadecane (Table S7). Such a broad catabolic potential is unusual among pollutant degrading bacteria, and therefore these strains should have special attention as they may have a beneficial adaptation and metabolic potential compared with the strains degrading only one type of hydrocarbon. Two strains, *Acinetobacter venetianus* ICP1 and *Pseudomonas oleovorans* ICTN13, which can degrade various aromatic pollutants and also aliphatic hydrocarbons (Table S7), were selected for the whole genome sequencing and growth experiments in the presence of pollutants to ascertain their metabolic capacity. These studies will be discussed in the next chapters.

LmPH was detected only in the strains belonging to the genus *Pseudomonas* (five strains) and *Acinetobacter* (two strains) that were able to grow on the phenol- and/or toluene-containing minimal plates (Table S7). For the cleavage of the catechol, all of the isolated phenol- and/or toluene-degrading *Pseudomonas* strains used catechol 2,3-dioxygenase, while the phenol-degrading *Acinetobacter* strains used catechol 1,2-dioxygenase (Table S7). Although strains from the same family were morphologically and phenotypically different, partial sequences of the determined catabolic genes were identical. However, the metagenomic approach with the three WWTP samples did not reveal any closely related sequences of the catabolic genes which were found in the isolated representative strains of *A. venetianus* ICP1 and *P. oleovorans* ICTN13 (Figures S1–S3).

The culture-dependent community composition analysis revealed that Gammaproteobacteria were dominating in the clarifier sample and *Bacilli* in the surge pond sample. The *Pseudomonas* and *Bacillus* strains were able to degrade cresols, but only the *Bacillus* strains were able to grow on Na-salicylate-containing minimal plates. The *Pseudomonas* strains ICTN13 and ICTN5 were able to also degrade toluene.

Crude oil processed in the refinery is drilled from the undersea oil reservoir, and therefore the wastewater also has higher salt concentrations. The salt tolerance of the strains isolated in this study ranged from 5% to 20%, whereas some strains (ISM2, ICM11, ICF1, ICA5 and ISHd4) were able to grow only if the medium contained at least 2.5% salt (Table S7).

### 3.4. Analysis of Genes of the Aromatic Compounds Degradation Pathways of the Strains ICTN13 and ICP1

**Figure 3:**
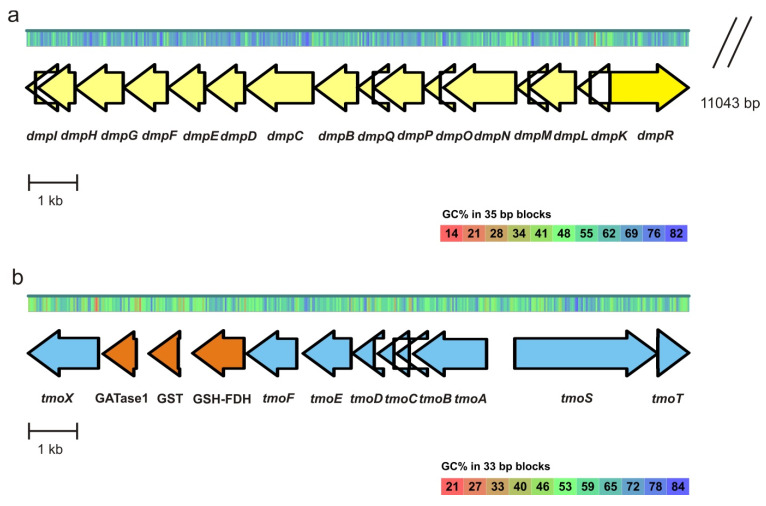
Genetic organization of the phenol degradation operon (*dmp*; GenBank accession number MK134569) and toluene degradation operon (*tmo*; GenBank accession number MK134568) of the *Pseudomonas oleovorans* ICTN13. (**a**) The genes of the *dmp* operon (yellow): *dmpI*, 4-oxalocrotonate isomerase; *dmpH*, 4-oxalocrotonate decarboxylase; *dmpG*, 4-hydroxy-2-oxovalerate aldolase; *dmpF*, acetaldehyde dehydrogenase; *dmpE*, 2-oxopent-4-dienoate hydratase; *dmpD*, 2-hydroxymuconic semialdehyde hydrolase; *dmpC*, 2-hydroxymuconic semialdehyde dehydrogenase; *dmpB*, catechol 2,3-dioxygenase; *dmpQ*, ferredoxin-like gene; *dmpKLMNOP*, components of phenol hydroxylase; *dmpR*, sigma-54-dependent Fis family transcriptional regulator. (**b**) The genes of the *tmo* operon (blue): *tmoX*, outer membrane protein coding gene, transport protein; *tmoF*, toluene-4-monooxygenase electron transfer component; *tmoE*, toluene-4-monooxygenase; *tmoD*, toluene-4-monooxygenase; *tmoC*, ferredoxin; *tmoB*, toluene-4-monooxygenase; *tmoA*, toluene-4-monooxygenase, *tmoST*, two-component signal transduction system. Genes inserted to the *tmo* operon (orange): GATase1, type 1 glutamine amidotransferase; GST, glutathione S-transferase; GSH-FDH, glutathione-dependent formaldehyde dehydrogenase.

The *Pseudomonas oleovorans* strain ICTN13 can grow on phenol, toluene and methylphenols (*p*-, *o*- and *m*-cresols; Table S7). The assembled draft genome of the strain ICTN13 is 5,240,773 bp, the N50 is 1,061,071 bp and it has six contigs. The average GC content of the genome is 64.65% and it contains 5213 genes. According to CheckM, the genome’s completeness is 100% and there is no detectable heterogeneity, but there is a 0.54% of contamination. The DNA sequence analysis of the genome of this strain revealed an operon (depicted in Figure 3a), which is structurally similar to the *dmp* operon of the *Pseudomonas* sp. CF600 (identity 75%–99%; [50]). The phenol degradation operon identified in the strain ICTN13 consists of genes encoding for a multicomponent phenol hydroxylase (mPH), catechol *meta* pathway, and a regulatory gene *dmpR* encoding for a protein related to the sigma-54-dependent Fis family of transcriptional regulators.

At close proximity, about 11 kb from the phenol degradation operon, operon carrying genes related to a toluene catabolic operon (*tol* operon) (Figure 3b) are located, which is structurally mosaic. This operon is partly similar to the toluene operon of the *Pseudomonas benzenivorans* DSM8628 (identity 96%–98%) and the *Pseudomonas mendocina* KR1 (identity 54%–70%) [51,52]. Similarly to benzene, toluene and the *p*-cresol degrading *P. benzenivorans* DSM8628 [53], three genes–glutamine amidotransferase, glutathione S-transferase and the glutathione-dependent formaldehyde dehydrogenase (Figure 3b)—are inserted between the *tmoX* and *tmoF* genes of the *tol* operon of ICTN13, sharing an identity of 94%–98%. Bacterial glutathione S-transferases are involved in the degradation of the different monocyclic aromatic compounds (toluene, xylenes, phenols, atrazine) and polycyclic aromatic hydrocarbons, and are involved in the protection against chemical and oxidative stress and antimicrobial drug resistance [54,55].

The *tol* operon of the strain ICTN13 contains, distinctly from the *P. benzenivorans* strain DSM8628, the genes encoding for TmoS and TmoT that in the *P. mendocina* KR1 (59% and 61% identity, respectively) have been shown to be involved in the regulation of the toluene 4-monooxygenase (T4MO) operon [51]. In the strain *P. mendocina* KR1, toluene is catabolized to *p*-cresol, which is stepwise oxidized to *p*-hydroxybenzoate and further degraded via the protocatechuate ortho pathway [51]. A genome-wide analysis revealed that the protocatechuate degradation pathway-encoding genes are absent in strains ICTN13 and DSM8628.

In the genome of the *Pseudomonas stutzeri* OX1 the toluene/o-xylene monooxygenase (ToMO)-encoding operon is located close to the phenol/*p*-cresol operon. Their substrate specificities overlap, but ToMO is more efficient in the degradation of non-oxidized substrates such as benzene, *o*-xylene and toluene, while phenol/cresol hydroxylase is more efficient in the oxidation of hydroxylated substrates such as cresols and phenol [56,57]. It has been suggested that ToMO catalyzes the oxidation of toluene to *p*-cresol that will be oxidized by the phenol/*p*-cresol hydroxylase [56,57], and the formed methylcatechols are catabolized via the catechol degradation meta pathway [58]. Some toluene monooxygenases, for example toluene 4- and 3-monooxygenases (T4MO and T3MO), hydroxylate toluene specifically at the ortho, meta or para positions [59,60] while the ToMO of the *P. stutzeri* OX1 has a relaxed toluene regiospecificity [61]. Sazinsky et al. (2004) have shown that the regiospecific hydroxylation is determined by the topology of the active site pocket [62]. Similar to the ToMO of the strain OX1, the toluene monooxygenase of the strain ICTN13 has at the active site position 180 Met residues but at the position 103, it is Ala instead of Glu. Although the hydrophobic side chain of the Ala residue is shorter when compared with that of the Glu residue, it has also a β carbon that has been proposed to be important in the relaxed regiospecificity of ToMO [62]. Based on this information, we can speculate that the toluene monooxygenase of the strain ICTN13 may also have relaxed regiospecificity. This hypothesis is supported by the ability of the strain ICTN13 to grow on *p-*, *m*- and *o*-cresols (Table S7), the products of the hydroxylation of toluene.

**Figure 4:**
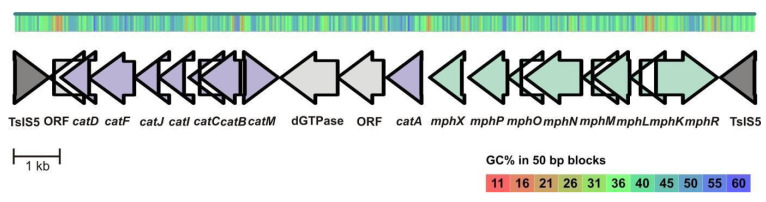
Genetic organization of the catechol degradation operon (*cat*) and phenol degradation operon (*mph*) of the *Acinetobacter venetianus* ICP1 (GenBank accession number MK140631). The genes of the *cat* operon (violet): *catD*, 3-oxoadipate enol-lactone hydrolase; *catF*, 3-oxoadipyl-CoA thiolase; *catJ*, 3-oxoadipate CoA-transferase subunit B; *catI*, 3-oxoadipate CoA-transferase subunit A; *catC*, muconolactone delta-isomerase; *catB*, muconate cycloisomerase; *catM*, LysR family transcriptional regulator; *catA*, catechol 1,2-dioxygenase. The genes of the *mph* operon (green): *mphX*, repressor; *mphKLMNOP*, components of phenol hydroxylase; *mphR*, sigma-54-dependent Fis family transcriptional regulator. TsIS5 (dark grey), IS5 family transposase.

In the case of the *Acinetobacter venetianus* strain ICP1, the draft genome is 3,604,535 bp, the N50 is 203,710 bp and it has 34 contigs. The average GC content of the genome is 39.08% and it contains 3515 genes. According to CheckM, the genome’s completeness is 100%, and there is 33.33% heterogeneity and 0.42% of contamination. Similar to the *P. oleovorans* ICTN13, the strain ICP1 has a multicomponent phenol hydroxylase for the degradation of phenol to catechol, but catechol is degraded via the ortho pathway (Figure 4). The phenol degradation operon (*mphXKLMNOPR*) of ICP1 is structurally similar to the operon described in the *Acinetobacter pittii* PHEA-2 (formerly *Acinetobacter calcoaceticus* PHEA-2) [63] and shares a 34%–81% identity. The catechol degradation genes (*cat*) in *cat* operon (Figure 4) are structurally similar to the *Acinetobacter* sp. ADP1 and share a 65%–76% identity [64].

In addition, the structures of the phenol catabolic operons of ICP1 and PHEA-2 are different from that of the phenol-degrading strain *A. calcoaceticus* NCIB8250, which has no *mphX* gene. It has been shown that *mphX* encodes for a transcriptional repressor protein which negatively affects the expression of the mPH genes [63]. It should be noted that most regulatory proteins of the aromatic catabolic pathways act as transcriptional activators and the regulation by the repressor is generally rare in aromatic compounds’ catabolism [65,66]. Although the exact regulatory mechanism of the *mphX* gene in PHEA-2 is not known, it was proposed that in the presence of phenol, MphX prevents the accumulation of catechol and cis,cis-muconate, which are toxic to the cells. However, experiments with the *mphX*-deletion mutant showed a faster phenol utilization than by the presence of the wild-type PHEA-2 operon [63].

Zhan and co-workers (2012) compared the genomes of three *Acinetobacter* species and declared that phenol degradation is not widely distributed among that genus [64]. Therefore, it is possible that these catabolic genes are obtained by a horizontal gene transfer. Indeed, in the strain ICP1, the *mph*-*cat* operons are located between two IS5 family transposases flanked by 16-nt long inverted repeats, indicating that this transposon was probably acquired through a horizontal gene transfer. Genes can become mobilized and dispersed across different strains, even to unrelated bacteria if they are flanked by two properly orientated identical IS elements [67].

In addition to the phenol hydroxylase cluster and the catechol degradation ortho pathway genes, the genome of the *A. venetianus* strain ICP1 contains the gene cluster (GenBank accession number MK134570) related to the protocatechuate degradation genes (named as the *pca* gene cluster) and the *qui* genes, encoding for the conversion of the hydroaromatic compounds quinate and shikimate into protocatechuate. This operon is structurally similar to that identified in the *Acinetobacter* sp. ADP1. However, the *pob* genes, encoding for the catabolism of *p*-hydroxybenzoate to protocatechuate, alongside the *pca-qui* region [68,69] are lost in the strain ICP1.

### 3.5. Comparison of the Growth of the Strains ICP1 and ICTN13 on Phenol

**Figure 5:**
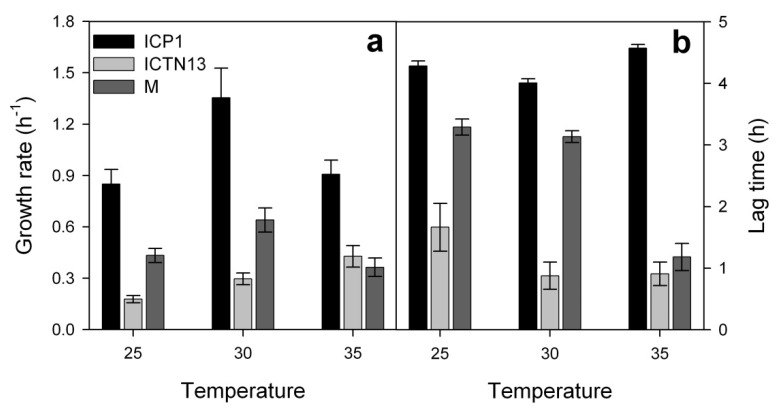
Growth rates (**a**) and lag-times (**b**) of the strains ICP1, ICTN13 and their mixture (M) cultivated in a minimal medium supplemented with 1.3 mM phenol and incubated at various temperatures (25, 30, and 35 °C). Error bars indicate standard deviation of the three independent experiments with three technical replicates.

**Figure 6:**
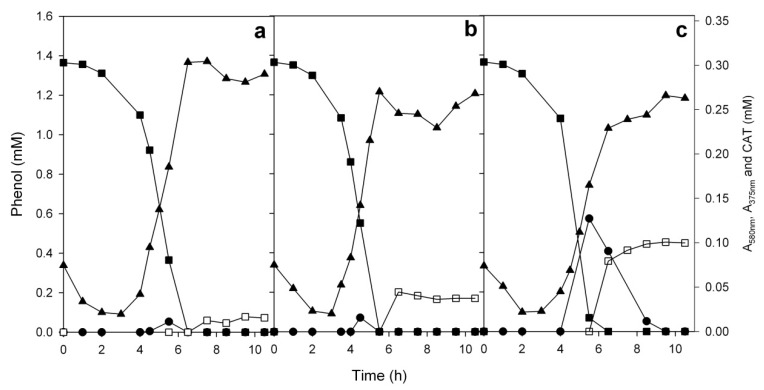
Growth of the strain ICP1 on 1.3 mM phenol incubated at 25 (**a**), 30 (**b**), and 35 (**c**) °C. Growth (▲, measured as absorbance at 580 nm), degradation of phenol (■) and accumulation of catechol (CAT, ●) and colored intermediate (□, measured as absorbance at 375 nm). Concentrations of the substrates and intermediates are the averages of triplicate determinations; the combined standard uncertainties of the results were between 2.1% and 3.4%.

**Figure 7:**
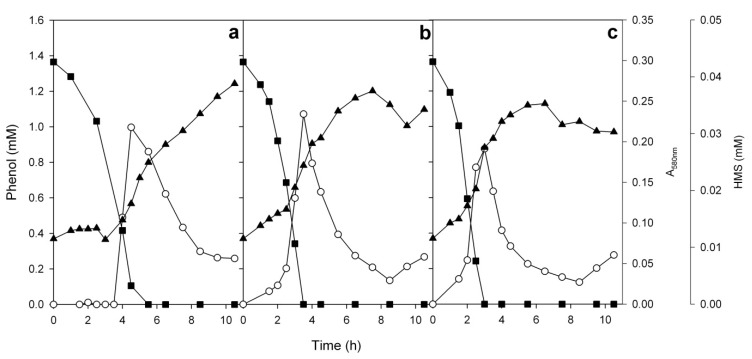
Growth of the strain ICTN13 on 1.3 mM phenol incubated at 25 (**a**), 30 (**b**), and 35 (**c**) °C. Growth (▲, measured as absorbance at 580 nm), degradation of phenol (■) and accumulation of 2-hydroxymuconic semialdehyde (HMS, ○). Concentrations of the substrates and intermediates are the averages of triplicate determinations; the combined standard uncertainties of the results were between 2.1% and 3.4%.

Phenol is one of the simplest aromatic compounds found in crude oil and can be used as a model substrate for the elucidation of the biodegradation ability of aromatic compounds. A metagenomic analysis of the samples of the refinery’s WWTP revealed the presence of diverse catabolic genes involved in the degradation of phenol. The strains ICP1 and ICTN13 degraded phenol (growth is observed on minimal plates containing up to 5 mM phenol) via different catechol degradation pathways and were therefore selected for the proper characterization. We examined the growth of these strains in a liquid phenol-containing (1.3 mM) M9 minimal medium and determined the effect of the temperature (25, 30 and 35 °C) on the growth of bacteria and measured the concentration of phenol and its degradation products in the growth medium (Figure 5, Figure 6 and Figure 7).

The optimum growth temperature for the *A. venetianus* ICP1 was 30 °C when the growth rate of the bacterium was the highest (Figure 5a and Figure 6b). At 35 °C, the growth rate was diminished and also the concentration of catechol determined in the growth medium was higher than that at the lower temperatures (Figure 6). The optimum growth temperature of the strain *Acinetobacter* sp. PD12 was also between 28–32 °C, and at higher temperatures, the growth rate diminished sharply [70]. These data are in quite good accordance with the results obtained with the *A. venetianus* strain ICP1. In the late-exponential growth phase, the growth medium of the strain ICP1 turned brownish (colour intensity was determined from the cell-free growth medium spectrophotometrically at 375 nm), probably due to the auto-oxidation of catechol to quinones. Notably, the colour was more intensive at higher temperatures (Figure S4). The colour of the quinones-containing solutions depends on the concentration of quinone, additives (e.g., metal ions), pH, etc. [71]. Here it is important to note that in the redox environment of biological systems, quinones may also cause toxicity via the formation of reactive oxygen species [71].

In contrast to the strain ICP1, the growth rate of the strain ICTN13 on 1.3 mM phenol was the highest at 35 °C, but it remained still 2 to 4.5 times lower than that of the strain ICP1 (Figure 5a). At the same time, the lag-time was shorter for the strain ICTN13 in comparison with the strain ICP1 (Figure 5b). During the growth of ICTN13 on phenol, the accumulation of 2-hydroxymuconic semialdehyde (HMS) was observed (Figure 7). After the accomplishment of the maximum concentration of this intermediate (similar at all used temperatures), the growth of the strain ICTN13 diminished. Later, after the depletion of phenol, the cells of the strain ICTN13 started to consume HMS from the growth medium, although its depletion was not complete (Figure 7).

The growth experiments on phenol revealed that although the strains ICP1 and ICTN13 were using different pathways for the degradation of phenol, in both cases, the intermediates accumulated in the medium. The results of our previous study indicated that in the case of the catechol meta pathway, the accumulation of HMS could cause the reduced level of the expression of the pathway due to the reduced level of the transcription of the C23O gene, and consequently, the accumulation of catechol [22]. This phenomenon was observed when the phenol- and salicylate-degrading *Pseudomonas pseudoalcaligenes* C70 was grown on 1.3 mM salicylate, but not on 1.3 mM phenol. As the determined concentrations of HMS in the current study were similar with that of the strain C70 [22], it is possible that the transcription of the C23Os of the different catabolic operons have different sensitivities to this accumulating intermediate.

The activity of the enzymes as well as the general cell physiology depends on the growth temperature of the bacteria. As the strains ICP1 and ICTN13 originate from India, where the average air temperature throughout the year is 24–30 °C, it was quite expected that both strains can grow at 35 °C. The results published by El-Naas et al. (2009) have shown that growth temperatures less than 30 °C caused a decrease in the bacterial activity and an increase in the inhibitory effect of phenol on the bacteria, especially at higher phenol concentrations (>0.8 mM) [72]. At the same time, they also suggested that the incubation temperatures higher than 35 °C may harm the key enzymes responsible for the degradation of phenol. This may be the reason why the accumulation of catechol in the phenol-containing growth medium of the strain ICP1 was increased by elevating the incubation temperature to 35 °C.

### 3.6. Growth of the Mixed Culture of the Strains ICP1 and ICTN13 on Phenol

**Figure 8:**
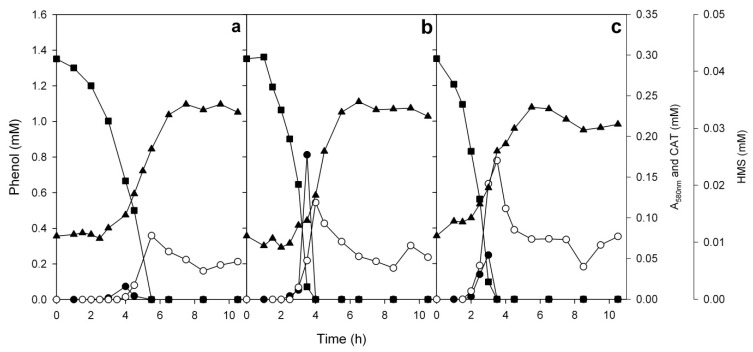
Growth of the mixed cultures of the strains ICP1 and ICTN13 on 1.3 mM phenol at 25 (**a**), 30 (**b**), and 35 (**c**) °C. Growth (▲, measured as absorbance at 580 nm), degradation of phenol (■), accumulation of 2-hydroxymuconic semialdehyde (HMS, ○) and catechol (CAT, ●). Concentrations of the substrates and intermediates are the averages of triplicate determinations; the combined standard uncertainties of the results were between 2.1% and 3.4%.

The degradation of pollutants in the WWTP is performed by diverse microbial communities whose compositions change through the treatment process, as we saw in the metagenomic analysis of the samples. The experiments with the single strains of ICP1 and ICTN13 on phenol revealed the accumulation of the intermediates of the phenol catabolic pathways. The growth parameters of these strains also differed. These findings led to the question of how is phenol degraded in the mixed cultures of the strains ICP1 and ICTN13 possessing different phenol degradation pathways? The degradation of phenol and the accumulation of the intermediates in the mixed culture of the strains ICP1 and ICTN13 are shown in Figure 8. Similar to the single strain cultivations, the accumulation of catechol and HMS was observed also in the mixed cultures. However, catechol was degraded from the growth medium faster than by the ICP1 grown alone (Figure 6 and Figure 8), and after 10 h incubation, the growth medium was slightly yellow due to the accumulation of HMS (Figure S5). At the growth temperatures 25 and 30 °C, the lengths of the lag-time were similar, but at 35 °C, the lag-time was shorter and its length was similar to that of the strain ICTN13 (Figure 5b). As already mentioned above, the optimum growth temperature of the strain ICP1 is 30 °C, whereas at a higher temperature, the degradation of phenol was slower and the detected concentrations of catechol were higher (Figure 6). This is probably the reason why the growth parameters of the mixed cultures at higher temperatures resembled those of the strain ICTN13, growing faster at 35 °C.

**Figure 9:**
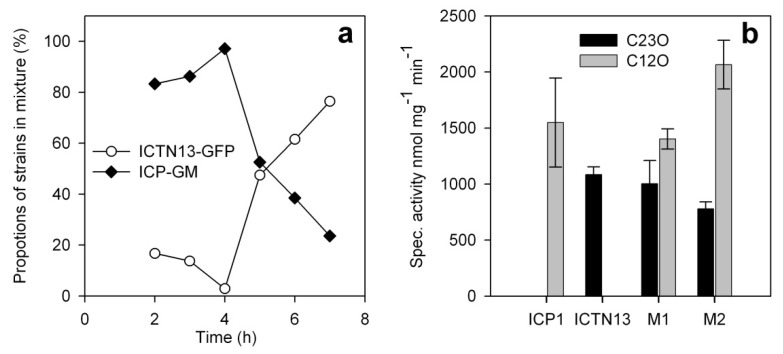
Proportions of the strains ICP1-GM (gentamicin-resistant strain ICP1) and ICTN13-GFP (GFP-labelled strain ICTN13) in the mixed cultures during the growth on 1.3 mM phenol at 30 °C (**a**), and specific activities of the key catabolic enzymes, C12O and C23O, in the crude extracts of the cells derived from the cultures either containing single strains or the mixture of these strains (M) sampled from the middle exponential (ICP1, ICTN13, M1 (5 h)) and early stationary growth phases (M2 (7 h)) (**b**). The growth curve of bacteria and the phenol utilization patterns were the same as presented in Figure 8b. The proportions of cells of both strains in the mixed cultures determined by the FACS analysis (Figure 9a) were taken into account in the calculation of the specific activities of the enzymes.

The proportion of the strains ICP1 and ICTN13 in the mixed cultures were determined by the FACS analysis of the cell populations sampled at different time points. To differentiate the two strains in the mixed cultures, the strain ICTN13 was tagged with the GFP marker. The details for the construction and detection of these strains are presented in the Materials and Methods section. We observed that although for the inoculation, equal amounts of the cultures of ICP1 and ICTN13 were mixed (based on the determination of absorbance at 580 nm), the FACS analysis of the lag-phase samples taken two hours after mixing the cultures of these strains revealed the predominance of the strain ICP1. However, an equilibrium between the two strains was achieved in the middle of the logarithmic growth phase (Figure 9a), when phenol and the produced intermediate, catechol, were consumed from the growth medium (Figure 8b). At the same time, the concentration of HMS, the intermediate of the catechol meta pathway, had reached the maximum value. Later the proportion of the strain ICTN13 increased, while the proportion of the strain ICP1 decreased in the mixed cultures (Figure 9a).

In order to further explore the expression level in the key enzymes of the catabolic pathways induced by phenol in the mixed cultures of the strains ICP1 and ICTN13, specific activities of C12O and C23O were determined in the samples taken during the middle exponential and early stationary growth phase of the bacteria (Figure 9b). The values obtained in both growth phases of the mixed cultures were similar to those when ICP1 or ICTN13 were grown alone. Slightly lower values were determined in the mixed cultures for the C12O activities in the middle exponential phase than in the early stationary phase.

The mixed culture cultivations on 1.3 mM phenol revealed the complementary impact of both strains. At the optimum growth temperature (30 °C), the length of the lag-time decreased and the growth rate increased, accompanied with diminished external concentrations of the intermediates of the phenol catabolism pathway. At the same time, the outcompeting of ICP1 by ICTN13 during the cultivation of bacteria in the phenol minimal medium could imply the different catabolic activities of these strains towards phenol. Also, we cannot exclude the possibility that a faster growth of ICTN13 could be achieved due to the utilization of catechol accumulated in the growth environment of the mixed cultures. Indeed, the studies with mixed cultures have shown that the accumulation of intermediates may stimulate the enrichment of bacteria that are able to metabolize these as substrates [73,74] and that the commensal interaction between the different species supports the degradation of pollutants [74]. To explain the phenomenon observed in the experiments with the mixed cultures in the current study, future experiments are needed to determine the kinetic parameters of phenol degradation and the growth parameters of the single strains at different incubation temperatures.

During the initial characterization of the isolated strains, we observed that both ICP1 and ICTN13 were able to grow on minimal agar plates containing up to 5 mM phenol. Compared with the growth of the bacterial colonies on agar plates, in the liquid cultures, the cells are more directly exposed to the toxic pollutant, and therefore the efficiency of the pollutant degradation could be lower. Therefore, in the next experiments, we determined the highest concentration of phenol in a liquid minimal medium by cultivating bacteria at the optimum growth temperature.

### 3.7. Effect of Higher Phenol Concentrations on the Growth of the Strains ICP1 and ICTN13

**Figure 10:**
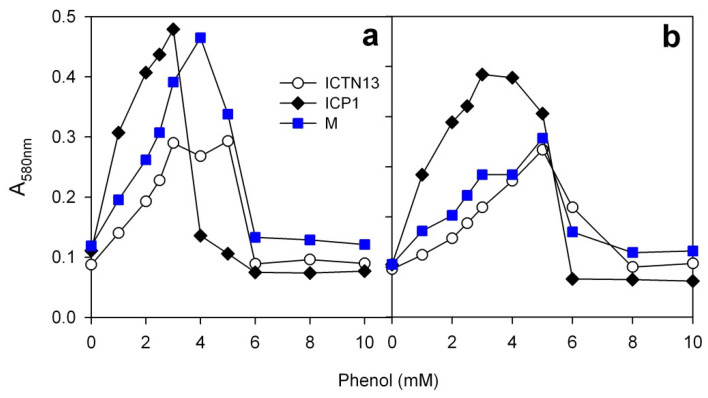
Estimation of the effect of the concentration of phenol on the growth of the strains ICP1 and ICTN13 and their mixture (M) on a minimal medium. The absorbance A_580 nm_ of the cultures was measured after the incubation of the bacteria at 24 h (**a**) and 48 (**b**) hours at 30 °C. Presented are the results of the three independent experiments each with at least three parallels. A statistical analysis of data is provided in the Supplementary Data S2.

The concentration of phenol in industrial wastewater may vary between 0.1 to 48 mM [49]. The initial phenol concentration has an important role in the biodegradation process because of the inhibitory effect on the activity of the microorganisms. To find out the maximum phenol concentration tolerated by the alternative catechol cleavage pathway-possessing strains ICP1 and ICTN13, when grown alone or in the mixed cultures, the growth experiments in a minimal medium supplemented with phenol (1.3 to 10 mM) were performed at 30 °C on multi-well plates (Figure 10).

After 24 h of the cultivation of the strain ICTN13 and the mixture of strains (M), the growth was detected in the wells containing up to 5 mM of phenol (Figure 10a). At the same time, the growth of the strain ICP1 was observed only in the wells containing up to 3 mM of phenol concentration (Figure 10a). However, after 48 h cultivation, we observed a statistically significant increase in the absorbance of the strain ICP1 also at higher phenol concentrations (4 mM and 5 mM) (Figure 10b, Supplementary Data S2). These experiments are in accordance with the finding that the strain ICP1 has a longer lag-time than the strain ICTN13 under the studied conditions (Figure 5b).

A statistically significant increase in the absorbance was determined between 24- and 48-h-incubated cultures of the strain ICTN13 on 6 mM phenol (Figure 10, Supplementary Data S2). We have previously shown that the length of the lag-time of the phenol-grown cultures of the pseudomonads-harboring multicomponent phenol hydroxylase and C23O is less affected by the initial phenol concentration, and these strains have higher inhibition constant values than the strains cleaving catechol via the ortho pathway [21]. In addition, the microcosm experiments with a mixture of such strains also revealed the predominance of the catechol meta pathway-possessing strain over the others during the growth in phenolic wastewater [74]. Thus, by drawing parallels between the results of these and the current studies, the better growth of the meta pathway-possessing bacteria at higher concentrations of phenol in comparison with the bacteria-degrading phenol via the ortho pathway might be a more general phenomenon.

There are several reports about the *Acinetobacter* strains able to degrade 5 mM or even up to 13 mM phenol [70,75]. Different from the results of the current study, these studies did not report an accumulation of intermediates. This might be a reason why the strain ICP1 grows slower at higher phenol concentrations. Here, it is important to mention that the growth of ICP1 was followed only for 48 h, while in the above discussed studies, the growth of the bacteria was observed over 9 days.

It should be noted that environmental pollutants can be both nutrients and stressors due to their toxic effects on cells that grow in contaminated sites [76]. The physiological response to stress may divert much of the metabolic energy that would otherwise be employed in the build-up of the catabolic pathways against pollutants. WWTPs treating refinery wastewater with biological methods contain microbial communities adapted to the degradation of crude oil components. The changing environmental conditions such as temperature, oxygen content, pH value and salinity affect the diversity and the structure of these communities [77]. Besides the bacteria that are able to degrade toxic compounds, also surviving in these raff conditions are strains producing surfactants, forming a biofilm, etc., that are essential in reducing the concentrations of toxic, recalcitrant and hydrophobic pollutants [78].

### 3.8. Comparison of Biofilm Formation Ability of the Strains ICP1 and ICTN13, and Their Mixture

Bacteria can grow in a liquid medium either planktonically, by forming aggregates, or by being attached to the surface to form a biofilm. The biofilm-forming bacteria have an advantage in the degradation of toxic compounds as in the biofilm, the biomass density is high, so bacteria interact with each other and share metabolites, etc., that results in an enhanced mineralization process. Therefore, biofilm-mediated bioremediation processes are also widely used in wastewater treatment. Coaggregation and biofilm formation has been shown to enhance the phenol tolerance [79,80,81].

**Figure 11:**
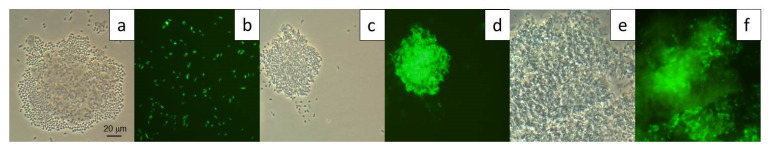
Microscope images of the strain ICP1-GM (**a**), ICTN13-GFP (**b**), and mixed cultures of the strains ICP1-GM and ICTN13-GFP (**c**–**f**) taken after 4 h (**c**, **d**) or 8 h (**a**, **b**, **e**, **f**) of growth on a minimal medium supplemented with 1.3 mM phenol. Images were made with a phase contrast (**a**, **c**, **e**) or with a fluorescence (**b**, **d**, **f**) microscope; image pairs—c, d and e, f—were made from the same field. Scale bar, 20 μm.

During the cultivation of the strains ICP1 and ICTN13 on the phenol-containing medium, we observed the formation of aggregates in flasks, especially in the case of the strain ICP1. Therefore, in order to explore the potential of the further application of the strains ICP1 and ICTN13 in the treatment of phenol-containing wastewater, we examined the ability of these strains to form aggregates and a biofilm when grown in the presence of phenol (Figure 11). The inspection of the samples derived from the 8-h-incubated cultures under the microscope revealed that the strain ICP1-GM formed aggregates (Figure 11a), while the cells of the strain ICTN13-GFP grew mainly planktonically and the cells were not adhered (Figure 11b). The 4-h cultures of the strain ICP1-GM also had aggregates, but they were smaller than that of the 8-h cultures.

In the mixed cultures, the aggregates were observed already after 4 h of cultivation, but in this case the coaggregation of the strains ICP1-GM and ICTN13-GFP was determined (Figure 11c,d). The results of the current study revealed that the cells of the strain ICTN13-GFP were present in the coaggregates with the strain ICP1-GM also after the 8-h incubation (Figure 11e,f), whereas the size of the aggregates increased with time (Figure 11c–f). All these results were in good accordance with our above-described observations that with the mixed culture of strains possessing different pathways, the degradation of phenol is more efficient than with the single strains.

**Figure 12:**
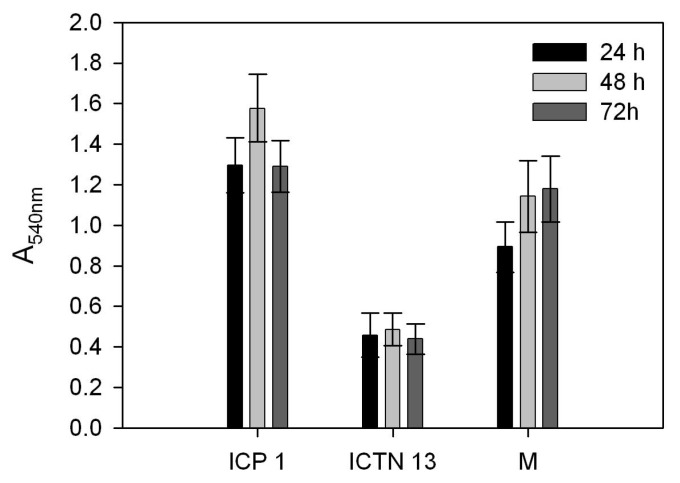
Biofilm formation on hydrophobic multi-well plates by the strains ICP1, ICTN13 and their mixture (M) in a minimal medium containing 1.3 mM phenol (PHE) after 24, 48, or 72 h of incubation. Biofilm formation was measured as the absorbance at 540 nm (A_540nm_) of the crystal violet solution rinsed from the cells that had adhered to the walls of multi-well plates after the corresponding incubation time. Error bars indicate standard deviation of the three independent biological experiments with 16 technical parallels.

In order to further examine the development of a biofilm of the single strains and their mixture, bacteria were grown in a minimal medium supplemented with 1.3 mM phenol on hydrophobic multi-well plates (Figure 12). The amount of biofilm formed to the walls of multi-well plates, quantified by the measurement of absorbance of the crystal violet solution (A_540nm_), was the highest in the cultures of ICP1. The formed biofilms were stable during the 72-h time frame.

The cell surface of the *Acinetobacter* species is highly hydrophobic and the adherence of the cells to the hydrophobic surfaces is high [82,83], which may be essential in the formation of a biofilm in biological wastewater treatment processes [84]. It has been shown that *Acinetobacter* strains do not only autoaggregate, but are able to coaggregate with other Gram-positive and negative bacterial species [85,86]. For example, a high adherence of the *Acinetobacter sp.* Tol5 to polyurethane foam was shown to be due to filamentous cell appendages, anchors and peritrichate fibrils [83]. An analysis of the genome of the phenol-degrading *A. pittii* PHEA-2 revealed the presence of genes encoding the type I fimbriae and type IV pili assembly proteins, and also the chaperon usher secretion system [64], which are all reported to be important for the attachment to surfaces and a biofilm formation [87]. All these genes are present also in the genomes of the strains ICP1 and ICTN13 (data not shown). It is believed that coaggregation may be facilitated by the receptors that are established by the functionally similar adhesins of the coaggregating strains [85,88]. Usually, the cells of biofilm-forming microbes are embedded in a matrix of extracellular polymeric substances (EPS), which are responsible for the structural and functional integrity of a biofilm. The composition of EPSs is diverse, depending on the bacterial strain and growth conditions. Pseudomonads are well known colonizers of different surfaces, and the mechanisms of adherence are the same [89,90] as for the previously described *Acinetobacter* strains. Coadherence of the *Acinetobacter* and *Pseudomonas* species has been observed in different environments. It has been shown that in mixed-species cultivations on benzyl alcohol, the cells of the *P. putida* KT2440 *wapH* mutant [91] and *P. putida* R1 [92] adhere to the microcolonies of the *Acinetobacter* sp. C6 that are cross-feeding the *Pseudomonas* cells with the excreted benzoate. These two *Pseudomonas* strains alone were not able to metabolize benzyl alcohol. A study by Christensen et al. (2002) suggested that *Acinetobacter* is effective in the first step of the degradation of benzyl alcohol but the next step, the degradation of benzoate, is slower [91]. At the same time, the capacity to metabolise benzoate is high in *Pseudomonas* strains and the external benzoate, released by *Acinetobacter* is also degraded. The biodegradation rate for aromatic compounds could be increased due to the close, mutually beneficial interactions amongst the cells in the biofilm. Thus, based on the results presented in the current study, we can speculate that in the mixed culture, phenol was degraded via the cooperation of two strains, whereas the strain ICP1 is responsible for the adherence and ICTN13 diminishes the accumulation of the toxic intermediates.

## 4. Conclusions

The current work established the relevant changes in the diversity and the distribution of the key catabolic proteins involved in the degradation of the aromatic hydrocarbons in the metagenomes of the microbial communities taken from the three different compartments of a wastewater treatment plant of an Indian crude oil refinery. The isolates obtained from the same samples revealed the prevalence of well-known aromatic compounds’ degrading bacterial genera such as *Pseudomonas*, *Acinetobacter* and *Bacillus*, among others. From the point of the operation of WWTPs and the improvement of the treatment efficiency, it would be useful to study the effect of seasons on the diversity and abundance of the bacterial communities in WWTPs. The sequencing of the whole genomes of the two isolates, *Acinetobacter venetianus* ICP1 and *Pseudomonas oleovorans* ICTN13, showed the potential of these strains to be efficient degraders of oil hydrocarbons as they are possessing genes for the catabolism of both aliphatic compounds and aromatic compounds. Monitoring the growth of bacteria in the phenol-containing minimal medium indicated that different catechol cleavage pathways expressing the strains ICP1 and ICTN13 in the mixed culture cultivations produced a lower number of toxic intermediates and exhibited a high phenol degradation efficiency. Further research is needed to determine the capability of these strains to degrade alkanes and complex mixtures, e.g., crude oil. Bacterial strains able to produce a biofilm and biosurfactant and exhibiting broad metabolic capabilities have the potential to be effective colonizers and degraders of oil-polluted environments. The proper study of such strains will help to understand their role in natural processes.

## Supplementary Materials

The following are available online at https://www.mdpi.com/2076-2607/8/5/652/s1, Figure S1: Phylogenetic analysis of the operational protein families (OPFs) of the phenol hydroxylases and related enzymes, by maximum likelihood method, detected by BLASTP from metagenomes of the WWTP of the crude oil refinery (sample C - yellow, sample G - red labels) and the isolated strains (green and blue labels), Figure S2: Phylogenetic analysis of the operational protein families (OPFs) of the catechol 2,3-dioxygenases, by maximum likelihood method, detected by BLASTP from metagenomes of the WWTP of the crude oil refinery (sample S - brown, sample C - yellow, sample G - red labels) and the isolated strain (blue label), Figure S3: Phylogenetic analysis of the operational protein families (OPFs) of the catechol 1,2-dioxygenases and related enzymes, by maximum likelihood method, detected by BLASTP from metagenomes of the WWTP of the crude oil refinery (sample C - yellow, sample G - red labels) and the isolated strain (green label), Figure S4: Picture of flasks taken after 10 h of growth of cell cultures of the strain ICP1 on 1.3 mM phenol incubated at 25, 30, and 35 °C, Figure S5: Picture of flasks taken after 10 h of growth of mixed cultures of the strains ICP1 and ICTN13 on 1.3 mM phenol at 25, 30, and 35 °C, Table S1: Overview of assembly statistics of shotgun metagenomics of samples taken from the surge pond sludge (S), from the clarifier outlet (C) and from the guard pond outlet (G) in January 2016, Table S2: Relative abundance of detected taxonomic assignments obtained from shotgun metagenomic dataset of surge pond sludge (S), the clarifier outlet (C) and the guard pond outlet (G) samples, Table S3: Diversity indexes of microbial communities of the surge pond sludge (S), the clarifier outlet (C) and the guard pond outlet (G) samples based on prokaryotic taxonomic assignments of shotgun metagenomic dataset, Table S4: The following query proteins domains were used for BLAST search against the assembled genomes, Table S5: Analysis of the detected protein sequences of phenol hydroxylase (PH), catechol 2,3-dioxygenase (C23O) and catechol 1,2-dioxygenase (C12O) identified in the assembled contigs by BLASTP in the surge pond sludge (S), the clarifier outlet (C) and the guard pond outlet (G) samples (in the protein code, the letter second of the last indicates the sample), Table S6: Relative abundance of operational protein families (OPFs)* of phenol hydroxylase (PH), catechol 2,3-dioxygenase (C23O) and catechol 1,2-dioxygenase (C12O) identified in the assembled contigs by BLASTP in the surge pond sludge (S), the clarifier outlet (C) and the guard pond outlet (G) samples, Table S7: Characterization of the aromatic compounds degrading strains isolated from the surge pond sludge (S) and the clarifier outlet (C) samples, Table S8: Bacterial strains and plasmids used in this study. Supplementary Data S1: Sequencing and analysis of the genomes of the isolated strains *Acinetobacter venetianus* ICP1 and *Pseudomonas oleovorans* ICTN13. Supplementary Data S2: Results of the statistical analysis for testing the effect of the phenol concentration on the growth of the strains ICP1 and ICTN13, and their mixture (M) in the minimal medium.
